# Give Your Titles

**Published:** 1881-11

**Authors:** 


### Selections.

### GIVE YOUR TITLES.

Says Dr. Billings, in his address before the International Congress: "The editors of transactions of societies, whether they are sent to journals or published in separate form, often commit numerous sins of omission in the matter of titles. The rule should be: that every article which is worth printing is worth a distinct title, which should be as concise as a telegram, and be printed in a special type. If the author does not furnish such a title, it is the editor's business to make it; and he should not be satisfied with such heading as, 'Clinical Cases,' 'Difficult Labor,' 'A Remarkable Tumor,' 'Case of a Wound, with Remarks.' The four rules for the preparation of an article for a journal will thus be: 1. Have something to say. 2. Say it. 3. Stop as soon as you have said it. 4. Give the paper a proper title."

We thus quote Dr. Billings as a hint to reporters of interesting matter. The busy reader and student opens a journal, and does not wish to spend time in finding out the contents of an article which, however interesting in itself, may be of no importance to this particular reader. So always give a title suggestive of the matter proposed to write about or the features of the case you are about to report.

